# Dimerized translationally controlled tumor protein increases interleukin-8 expression through MAPK and NF-κB pathways in a human bronchial epithelial cell line

**DOI:** 10.1186/s13578-018-0214-6

**Published:** 2018-02-20

**Authors:** Heewon Lee, Kyunglim Lee

**Affiliations:** Graduate School of Pharmaceutical Sciences, College of Pharmacy, Ewha Womans University, Seoul, 120-750 Korea

**Keywords:** BEAS-2B, Dimerized TCTP, Interleukin-8, MAPK, NF-κB

## Abstract

**Background:**

Histamine releasing factor (HRF) is a unique cytokine known to regulate a variety of immune cells in late allergic reactions. In the previous study, we revealed that the biologically active form of HRF is the dimerized translationally controlled tumor protein (dTCTP) for the first time, and confirmed the secretion of IL-8 cytokine by dTCTP in human bronchial epithelial cells. However, the signaling pathway by which dTCTP promotes the secretion of IL-8 is not known.

**Results:**

When the cells were stimulated with dTCTP, the canonical NF-κB pathway and ERK, JNK and p38 MAPK become activated. dTCTP promoted transcription of IL-8, which involved NF-κB and AP-1 transcription factors. NF-κB was found to be essential for the transcriptional activation of IL-8, while AP-1 was partially responsible for the transcriptional activation by dTCTP. p38 MAPK was found to be involved in post-transcriptional regulation of dTCTP by stabilizing IL-8 mRNA.

**Conclusions:**

This study demonstrated that dTCTP induces IL-8 secretion in BEAS-2B cells through transcriptional and post-transcriptional regulation of MAPK and NF-κB pathways. This study provides insight into the mechanism by which dTCTP induces inflammation.

**Electronic supplementary material:**

The online version of this article (10.1186/s13578-018-0214-6) contains supplementary material, which is available to authorized users.

## Background

Airway epithelium is not only the target of the inflammatory response, but it is also an important effector tissue that controls secondary mediators. It is the first tissue to encounter inhaled allergens and contributes to endogenous defense by regulating mucus secretion and ciliary movement. Airway epithelial cells are known to secrete various cytokines and chemokines under external stimuli. Interleukin-8 (IL-8) previously known as neutrophil chemotactic factor is one of them. IL-8 secretion is increased by oxidant stress, which thereby cause the recruitment of inflammatory cells and induces a further increase in oxidant stress mediators, making it a key parameter in localized inflammation [1].

Histamine releasing factor (HRF) was first described as a substance capable of histamine release from basophils present in the secretion of activated mononuclear cells [2]. Its clinical importance has been emphasized since it was found in the biological fluids of allergic patients. In particular, the fact that it was found in the sera of asthmatic patients and bronchoalveolar lavage fluids of airway inflamed mice suggests its role in airway inflammatory diseases. In a previous report, amino-terminal sequencing led to the identification of Translationally controlled tumor protein (TCTP) as the same as HRF [3]. TCTP, a highly conserved and ubiquitously expressed protein in all eukaryotic cells, has been reported to be involved in a variety of cellular processes such as cell growth and development [4]. However, since not all TCTP secreted by cells exhibited histamine releasing activity, the relationship between HRF and TCTP remained unclear.

Our previous studies demonstrated that the biologically active form of TCTP is its dimeric form of TCTP (dTCTP) and that dimerization is essential for generating its cytokine-like activity of TCTP [5]. The BEAS-2B cell line derived from human normal bronchial epithelium was the first non-immune cell of HRF target cells reported. When oxidative stress was applied to BEAS-2B cells, HRF was secreted and extracellular HRF promoted the release of inflammatory cytokines such as IL-8 and GM-CSF [6], suggesting the presence of dTCTP-specific receptors on the BEAS-2B cell line.

In this study, we investigated the IL-8 secretion signaling pathways by dTCTP in BEAS-2B cells and demonstrated that in BEAS-2B cells, dTCTP induces IL-8, an inflammatory cytokine, through MAPK and NF-κB signaling.

## Methods

### Cell culture

Human bronchial epithelial cells, BEAS-2B, were purchased from the American Type Culture Collection (ATCC, CRL-9609) and cultured in bronchial epithelial cell growth medium (BEGM, Lonza) at 37 °C and 5% CO_2_. Airway Epithelial Cell Growth Medium (Promocell, C-21060) was used in small interfering RNA (siRNA) transfection experiments.

### Reagents and antibodies

The sources of reagents and antibodies used in this study are as follows: IPTG was from Duchefa (Haarlem, The Netherlands); Ampicillin sodium salt and chloramphenicol were from USB (OH, USA); Penicillin–Streptomycin was from Thermo Fisher Scientific (UT, USA). MAPK Inhibitors PD98059, SP600125, SB203580 and BAY11-7082 were from Calbiochem (CA, USA). The oligonucleotides used in these experiments were synthesized by Bioneer (Seoul, Korea). Antibodies against phospho-p44/p42 MAPK (Thr202/Tyr204), p44/p42 MAPK, phospho-SAPK/JNK (Thr183/Tyr185), SAPK/JNK, phospho-p38 MAPK (Thr180/Tyr182), p38 MAPK, phospho-MAPKAPK-2 (Thr222), MAPKAPK-2, phospho-IκBα (Ser32/36), IκBα, Lamin A/C and β-actin were from Cell Signaling Technology (MA, USA); GAPDH antibody was from AbFrontier (Seoul, Korea); NF-κB (p65) antibody was from Enzo Life Sciences (NY, USA).

### Recombinant protein production

We prepared the recombinant proteins as previously described [5]. Briefly, pRSET A/Del-N11 TCTP was transformed to *E. coli* strain BL21(DE3)pLysS and cells were grown at 37 °C and 220 rpm in Luria–Bertani medium containing the 100 μg/ml ampicillin and 34 μg/ml chloramphenicol. The pre-culture medium was diluted 1:100 with 400 ml and cultured until OD_600_ reached 0.6–0.8 (Hitachi, U-3000). After IPTG was added to a final concentration of 0.4 mM, the culture was incubated at 37 °C and 200 rpm for 2 h 30 min. Cells were harvested by centrifugation at 7140×*g* (Sorvall, SLA-3000 rotor) for 10 min at 4 °C and stored at − 70 °C or used directly. Cell pellets were resuspend in ice-cold equilibration buffer (50 mM sodium phosphate, 300 mM NaCl, 10 mM imidazole; pH 7.4) with 1 mM PMSF and disrupted by sonication (Kyung Ill, KTA-400). Sonication was performed three times on ice for 30 s at 1/10 of maximal amplitude followed by centrifugation for 40 min at 12,000×*g* (Sorvall, SS34-rotor).

The supernatants containing soluble proteins were purified with HisPur™ Cobalt Resin (Thermo, 89965) according to the manufacturer’s instruction. Subsequently, the proteins were eluted and the mixtures desalted using a PD-10 desalting column (GE Healthcare, 17-0851-01) and loaded onto HiTrap Q HP column (GE Healthcare, 17-1153-01) which was equilibrated with buffer A (20 mM Tris, 1 mM EDTA, 50 mM NaCl; pH 7.4). Proteins were separated by AKTA FPLC systems (GE Healthcare) and eluted with buffer B (20 mM Tris, 1 mM EDTA, 1 M NaCl; pH 7.4) with a constant flow rate of 1 ml/min. The eluted fractions were separated by SDS-PAGE and the samples containing dTCTP were collected and desalted with PBS. Purified dTCTP was concentrated using Vivaspin 500 (Sartorius, VS0122) and stored at − 70 °C until use.

### Measurement of IL-8

BEAS-2B cells were seeded at 4000 cells per well in 48-well plates (Nunc). When the cells became 60% confluent, they were washed twice with 1% penicillin–streptomycin/BEBM, and treated with or without inhibitor for 30 min at indicated concentrations followed by 10 μg/ml dTCTP. After 16–20 h, IL-8 in the media was measured with Legend MAX™ Human IL-8 ELISA Kit (BioLegnd, 431508) according to the manufacturer’s protocol.

### Immunofluorescence confocal microscopy

Sterilized cover slips were coated with poly-d-lysine (Sigma) in 24 well plates (Nunc) for 2 h 30 min and BEAS-2B cells were seeded at 35,000 cells per well. After 36–48 h, cells were serum starved for 2 h and treated with PBS or 10 μg/ml dTCTP for 1 h followed by fixing with 4% paraformaldehyde (Sigma, P6148) in PBS for 15 min at room temperature. Cells were permeabilized with 0.2% Triton X-100 in PBS for 5 min on ice and blocked with 1% bovine serum albumin in permeabilization buffer for 1 h at room temperature. Rabbit anti-NF-κB (p65) antibody (Enzo, ALX-210-574) was diluted 1:300 with blocking buffer and treated to the cells overnight at 4 °C. The stained cells were washed with PBS and probed with Alexa Fluor 488-conjugated goat anti-rabbit IgG (Invitrogen, A11008) diluted 1:1000 in blocking buffer for 30 min at room temperature in a shaded chamber. After washing with PBS, cells were counterstained with DAPI using ProLong™ Gold Antifade Mountant (Invitrogen, P36931). Confocal microscopy was performed using a Carl Zeiss Laser Scanning Systems LSM 510.

### Immunoblotting

BEAS-2B cells were washed with PBS 3 times and harvested by scraping with ice-cold lysis buffer (50 mM Tris–HCl; pH 7.4, 150 mM NaCl, 1 mM EDTA, 0.25% deoxycholate, 1% Triton X-100) containing protease inhibitor cocktail (Roche, 11 836 170 001) and phosphatase inhibitor cocktail (Sigma Aldrich, P5726 and P0044). Cells were vortexed thoroughly for 30 s and set on ice for every 10 min. After 30 min, lysates were centrifuged at 12,000×*g* for 20 min at 4 °C, and the supernatants were collected. Protein contents were quantified by Bradford assay (Bio-Rad, CA, USA) and 5–15 μg of each sample were separated by SDS-PAGE, transferred to PVDF membranes and probed with primary antibodies followed by incubation with HRP-conjugated goat anti-mouse/rabbit IgG antibody (Bio-rad, CA, USA). Proteins of interest were visualized with chemiluminescent sensitive plus HRP substrate (Surmodics, LERI-0110-2C) and detected with LAS-3000 (Fujifilm, Tokyo, Japan).

### Reverse transcription-PCR

Total RNA was isolated from cells using RNeasy Mini Kit (Qiagen, 74104) as described by the manufacturer. The isolated RNA had an A_260/_A_280_ ratio of 2.0–2.1 and 1 μg of each RNA samples were reverse transcribed into cDNA using High capacity cDNA Reverse transcription Kit (Applied Biosystems, 4368814). 1 μl of the resulting cDNA, 10 pmol of each forward and reverse primer, and the DNA polymerase mixture provided in AccuPower PCR PreMix (Bioneer, K-2016) in 20 μl reaction volume were amplified using PCR. The primers were synthesized by Genotech Co. Ltd. (Korea) and the sequences were: IL-8, 5′-CATGACTTCCA AGCTGGCCGTG-3′ (forward) and 5′-TCACTGATTCTTGGATACCACA GAG-3′ (reverse) and β-actin, 5′-CAGCTCGTAGCTCTTCTCCA-3′ (forward) and 5′-CAGCTCGTAGCTCTT CTCCA-3′ (reverse). The reaction mixtures were pre-denatured by incubating at 94 °C for 5 min followed by 27 cycles (for IL-8) or 30 cycles (for β-actin) of 94 °C for 30 s, 55 °C for 30 s and 72 °C for 30 s, and for final extension, they were incubated at 72 °C for 10 min. 8 μl of the amplified products were resolved on a 1% agarose gel containing SYBR™ Safe DNA gel stain (Invitrogen). Images were taken using E-Graph AE-9000 (Atto, Japan).

### Real-time quantitative PCR

Changes in IL-8 transcripts were confirmed by real-time PCR in cells treated with various doses of dTCTP. Levels of IL-8 mRNA was analyzed using TaqMan Gene Expression Assays (Applied Biosystems, 4331182) (Assay IDs: IL-8, Hs00174103_m1; GAPDH, Hs99999905_m1) according to the manufacturer’s protocol. 2 μl of cDNA was used for 20 μl reaction, and each samples were run in triplicates. Reaction mixtures were amplified with an initial denature step at 95 °C for 10 min, followed by 40 cycles of 95 °C for 15 s and 60 °C for 1 min using Applied Biosystems 7300 Real-Time PCR System (Applied Biosystems, ABI 7300). The expression of IL-8 mRNA was normalized to GAPDH mRNA. The efficiencies of the reactions were calculated by a fivefold serial dilution and estimated to be 90–95%.

### Luciferase assay

BEAS-2B cells were plated at 6 × 10^4^ cells/well into 96 well culture plate (Nunc). After 48 h at 70% confluency, BEAS-2B cells were cotransfected with 1 μg of experimental reporter plasmid along with 0.1 μg of control plasmid linked to the Renilla luciferase vector (pRL-TK, Promega) by using Lipofectamine 2000 reagent (Invitrogen). Experimental reporter vectors regulated by a synthetic promoter containing direct repeats of consensus sequences for AP-1 and NF-κB designated as pAP-1-Luc and pNF-κB-Luc were purchased from Stratagene. IL-8 promoter reporter plasmids with and without a mutation of NF-κB (GGATTTCCT to TAACTTTCCT) and AP-1 (TGACTCA to TATCTCA) binding site cloned into the pGL3-Basic vector, designated as pIL8, pIL8mutNF-κB, and pIL8mutAP-1 were kindly provided by HY Lee (Yonsei University, Korea) [7]. At 24 h after transfection, cells were washed and stimulated with dTCTP for indicated times and stored at − 70 °C. The luciferase activity of firefly and Renilla luciferase were measured using Dual-Luciferase Reporter Assay System (Promega, E1910) and read by a microplate luminometer (Berthold Technologies, MicroLumat Plus). The frozen cells were lysed with 30 μl of passive lysis buffer and gently rocked at room temperature until complete lysis was confirmed by microscopy. After transferring 20 μl of the cell lysate to a 96-well white luminometer plate, the activities of firefly and Renilla luciferases were measured sequentially form a single sample. The results were presented as the relative ratio of experimental vector to control vector.

### Electrophoretic mobility gel shift assay (EMSA)

BEAS-2B cells grown in 100 mm culture dish (Nunc) were serum starved for 2 h and stimulated with either PBS or dTCTP. The nuclear extracts were prepared using NE-PER™ Nuclear and Cytoplasmic Extraction Reagents (Thermo, 78833) according to the manufacturer’s manual. The protein contents of nuclear extracts were quantified, aliquoted, and used directly or stored at − 70 °C until use.

The sequences of the sense-strand oligonucleotides used in these experiments were as follows: NF-κB, 5′-AGTTGAGGGGACTTTCCCAGGC-3′, AP-1, 5′-CGCTTGATGAGT CAGCCGGAA-3′ (Promega Corporation, USA), mutant NF-κB, 5′-AGTTGAGGTAA CTTTCCCAGGC-3′, and mutant AP-1, 5′-CGCTTGATATGTCAGCCGGAA-3′. The complementary pairs of oligonucleotides were annealed using a heating block. The pair of nucleotides were mixed at a molar ratio 1:1, incubated at 95 °C for 5 min, and allowed to stand overnight at room temperature.

EMSA was performed as previously described with some modification. Briefly, 6% polyacrylamide gel was pre-run for 40 min in 0.5× TBE buffer. During this time, binding reactions were performed by adding 5 μg of nuclear extracts (as 2–4 μl of NE-PER) in 5× binding buffer (20% Glycerol, 5 mM MgCl_2_, 2.5 mM EDTA, 2.5 mM DTT, 250 mM NaCl, 50 mM Tris, 2.5 mg/ml poly dI·dC). Then 3′ end-biotinylated double stranded DNA was added at a final concentration of 1 pmol and incubated at room temperature for 20 min. The specificity of the binding reaction was confirmed by pre-incubating the nuclear extracts with tenfold molar excess of unlabeled DNA. The reaction mixtures were electrophoresed and transferred to nylon membrane (Roche, 11 209 299 001) at 4 °C. The membrane was cross-linked for 10 min using E-Graph AE-9000 (Atto Incorporation, Japan) equipped with 312 nm UV transilluminator. The biotin-labeled DNA on the membrane was detected using LightShift Chemiluminescent EMSA Kit (Thermo, 20148) according to the instructions.

### Small interfering RNA (siRNA)

BEAS-2B cells were seeded in 12 well plates (160,000 cells/well) and incubated for 24–36 h in growth media. At 60–65% confluency, cells were transfected with AccuTarget™ FAM labeled for Negative Control (Bioneer, SN-1021) or siRNA targeting p65 mRNA (Bioneer, 1128171) using Lipofectamine^®^ RNAiMAX Reagent (Invitrogen) at a final concentration of 20 nM in Opti-MEM^®^ (Thermo). After 6 h, media were changed with growth media and incubated for another 18 h. 24 h post-transfection, the transfection efficiency was confirmed by fluorescence microscopy (data not shown) and the cells were treated with or without 10 μg/ml of dTCTP for 16–20 h. The resulting supernatants were harvested and analyzed for IL-8 contents.

### Statistical analysis

Data are presented as means ± standard errors. Data were analyzed with GraphPad Prisms 5 software (GraphPad Software Inc., CA, USA). Statistical significance was determined using Student’s two-tailed unpaired *t* test for comparisons between two groups. For more than 3 groups, one-way ANOVA analysis was performed.

## Results

### dTCTP-induced IL-8 secretion is transcriptionally regulated

**Fig. 1:**
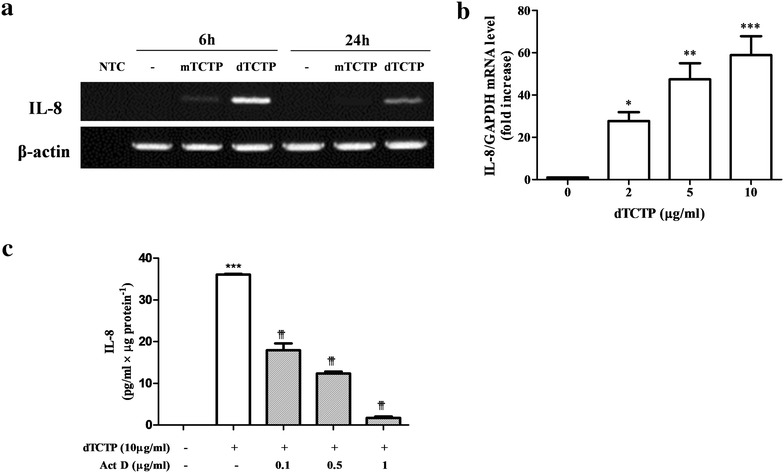
IL-8 induction by dTCTP is transcriptionally regulated. **a** BEAS-2B cells were incubated with 10 μg/ml of dTCTP for 6 or 24 h, and expression of IL-8 mRNA and β-actin mRNA was determined by RT-PCR. Data shown are representative of three independent experiments. **b** BEAS-2B cells were treated with indicated concentrations of dTCTP for 6 h. Levels of IL-8 mRNAs were measured using TaqMan-based-real time PCR, normalized to levels of GAPDH mRNA, and expressed as fold increases over control. Values are expressed as fold changes (mean ± SEM, n = 3). *p < 0.05, **p < 0.01 and ***p < 0.001, significantly different from untreated group. **c** BEAS-2B cells were pretreated for 1 h with 0.1–1 μg/ml of actinomycin D, and coincubated with 10 μg/ml dTCTP for an additional 24 h, followed by assay for IL-8 of the supernatants. Data shown are the mean with SEM of three independent experiments. ***p < 0.001 compared with bar 1 and ^†††^p < 0.001 compared with bar 2

Previously, Kim et al. showed that recombinant dTCTP, but not monomer TCTP (mTCTP), induced IL-8 secretion in BEAS-2B cells [5]. We analyzed IL-8 mRNA by RT-PCR in BEAS-2B cells which were treated with either mTCTP or dTCTP for 6 or 24 h. As shown in Fig. 1a, IL-8 mRNA was significantly induced in response to dTCTP treatment for 6 h. After 24 h, IL-8 mRNA decreased. However, when stimulated with mTCTP, BEAS-2B cells did not generate IL-8 mRNA at both time points. Changes in IL-8 transcripts were confirmed by real-time PCR in cells treated with various doses of dTCTP. Figure 1b shows that the IL-8 transcripts increased in a dose-dependent manner. During 6 h incubation with 2, 5 or 10 μg/ml of dTCTP, IL-8 transcripts were increased to mean ± SD of 27.7 ± 7.4, 47.4 ± 10.8 or 58.9 ± 15.5-fold respectively, indicating that dTCTP had specific action on IL-8 gene expression. Next, we investigated whether IL-8 induction by dTCTP is affected by actinomycin D (Fig. 1c). Cells stimulated with dTCTP for 6 h were treated with actinomycin D for 1 h and the resulting IL-8 protein was measured. When treated with actinomycin D at a low dose of 0.1 μg/ml, IL-8 protein expression was reduced to 49.7% of the control. In addition, the amount of IL-8 protein decreased in a actinomycin D concentration-dependent manner. These results suggest that dTCTP induces IL-8 by promoting the transcription of IL-8 gene.

### dTCTP activates transcription factor NF-κB and AP-1 in BEAS-2B cells

Since dTCTP appeared to upregulate transcription of IL-8 gene, we explored the transcription factors potentially involved in dTCTP-induced IL-8 production. The proximal promoter region of − 1 to − 133 bp is known to be sufficient for the expression of IL-8 [8, 9], which contains NF-IL6 (also known as C/EBPβ) transcription factor binding site as well as NF-κB and activator protein-1 (AP-1) binding sites. In addition, C/EBP homologous protein (CHOP) was reported to be a novel signaling mechanism mediating IL-8 release from activated T lymphocytes [10] and from cystic fibrosis bronchial epithelial cell lines [11]. Although not widely studied, it has been also suggested that cyclic AMP-responsive element binding protein (CREB) transcription factor may be involved in the regulation of the CXC chemokine [12]. Using in silico analysis, Bezzerri et al. [13] identified four transcription factor binding sites in the proximal region of the IL-8 promoter in human bronchial epithelial IB3-1 cell. The consensus sequences for NF-κB (− 80/− 72 bp), NF-IL6 (− 93/− 84 bp), AP-1 (− 126/− 120 bp), and CREB (− 171/− 164 bp) were mapped.

However, among those transcription factors implicated in IL-8 gene expression, NF-κB and AP-1 are most well-known key regulators for IL-8 gene expression in airway epithelial cells (reviewed in [14]). And specific elements involved in the regulation of NF-κB and AP-1 pathways have been reported to be different depending on the cell type and stimulus.

**Fig. 2:**
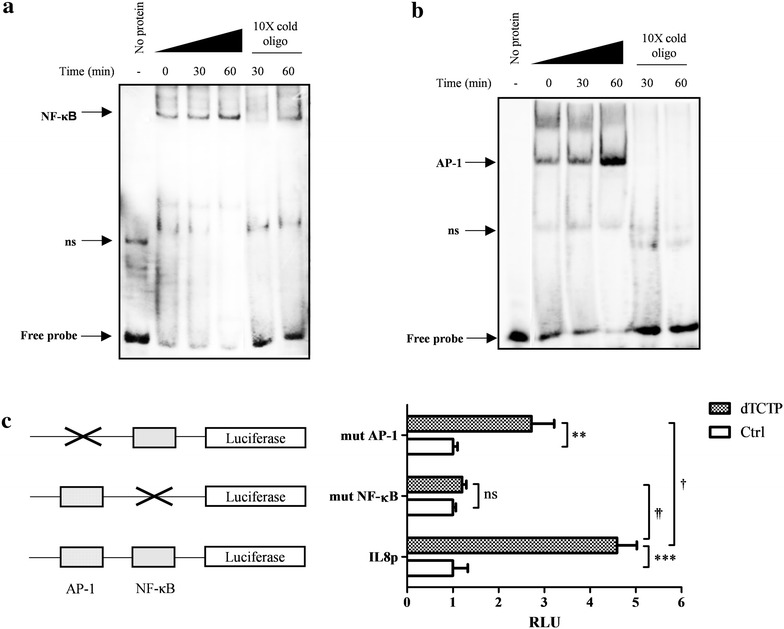
The role of NF-κB and AP-1 in dTCTP-induced IL-8 production. Electrophoretic mobility shift assays to measure the DNA-binding activities of NF-κB (**a**) and AP-1 (**b**) binding to their recognition sites were performed. Nuclear extracts were prepared from the BEAS-2B cells stimulated with 10 μg/ml of dTCTP for various time periods (0, 30 and 60 min). EMSA was performed using nuclear extract binding buffer (100 mm Tris, 500 mm KCl and 10 mm DTT; pH 7.5), and biotinylated probes were incubated at room temperature for 20 min. The protein–DNA complexes were electrophoresed on 6% polyacrylamide gels at 4 °C in ×0.5 TBE buffer and transferred to a nylon membrane. The UV cross-linked membrane was treated with streptavidin–horseradish peroxidase conjugate and then detected with CCD camera imaging device using the LightShift Chemiluminescent EMSA Kit. Unlabelled oligonucleotides were included to examine binding specificity. **c** Schematic presentation of IL-8 promoter luciferase reporter system and two mutant IL-8 promoters with an altered NF-κB or AP-1 binding site (left panel). Luciferase activities of BEAS-2B cells carrying IL-8 promoter reporter plasmids were measured using dual luciferase assay system. BEAS-2B cells were transiently transfected by Lipofectamine™ 2000 reagent with 1 μg of each IL-8 luciferase plasmids and 0.1 μg of pRL-TK. At 24 h after transfection, the cells were stimulated for 24 h with dTCTP (10 μg/ml). Values are expressed as fold changes (mean ± SEM, n = 3). **p < 0.01, ***p < 0.001; control vs dTCTP, ^†^p < 0.05, ^††^p < 0.01; dTCTP treated wild type vs mutant

To investigate whether NF-κB and AP-1 transcription factors are involved in dTCTP-induced signaling pathway, nuclear extracts from BEAS-2B cells stimulated with dTCTP for 0, 30 and 60 min were analyzed by EMSA using probes for NF-κB (Fig. 2a) and AP-1 (Fig. 2b). When the transcription factor complexes with the corresponding sequence, the band shifts due to the increase in the molecular weight. In both cases, the free probes were reduced by the addition of nuclear extracts and the DNA–protein complex increased most at 60 min after dTCTP treatment. This suggests that the nuclear extracts contain proteins that can bind the NF-κB and AP-1 consensus sequences. The DNA binding specificity of the protein was confirmed by the adding a tenfold excess of cold unlabeled oligonucleotide to the mixture of DNA and nuclear extracts. The cold probe competes with the labeled DNA probe to bind to the proteins, so that the intensity of the shifted band is reduced or eliminated. The binding specificity was also confirmed using a tenfold molar excess of unlabeled binding site mutant oligonucleotide that did not affect the formation of protein-DNA complexes (see Additional file 1: Figure S1).

These results demonstrated that dTCTP induces DNA binding of NF-κB and AP-1 transcription factors. Hoffmann et al. [14] revealed that the binding of p65 NF-κB to the endogenous IL-8 promoter contributed to the initiation of the transcription process by recruiting RNA polymerase II within 30 min upon IL-1 stimulation. The specific mechanism of transcriptional regulation of the IL-8 gene by the AP-1 dimer is not known at the atomic level, but is believed to contribute to an environment suitable for transcription initiation by complexing with other proteins. Therefore, EMSA results suggested that dTCTP can initiate IL-8 transcription via NF-κB and AP-1.

### NF-κB and AP-1 are required for IL-8 induction by dTCTP

To verify whether two candidates, NF-κB and AP-1, have a role in dTCTP-induced IL-8 expression, we used luciferase reporter gene designed to be driven by IL-8 promoter (Fig. 2c). Specifically, IL-8 promoter which was ligated into pGL3-Basic vector, designated as pGLIL8p, was transiently transfected to BEAS-2B cells for 24 h, and stimulated with dTCTP for 18 h. As a surrogate marker for IL-8 promoter activity, luminescence from lysed cells was measured. We compared luciferase activity of pGLIL8p transfected cells with that of pIL8pmutAP-1 and pIL8pmutNF-κB transfected cells, which have a mutation in binding sites for AP-1 and NF-κB, respectively. In cells introduced with pGLIL8p, dTCTP elevated luciferase activity to 4.6 fold over non-stimulated condition. However, defect in κB binding sites significantly impaired luciferase expression driven by IL-8 promoter, implying that IL-8 induction is predominantly mediated by NF-κB transcription factor. Without NF-κB, dTCTP did not enhance IL-8 transcription. However, cells transfected with pIL8mutAP-1 showed reduced promoter activity by 59.3%, but the reduction was not as significant as that in mutation with NF-κB. In the absence of activation process through AP-1, dTCTP enhanced IL-8 transcription 2.7-fold. These results show that both NF-κB and AP-1 are involved in the transcription of IL-8 by dTCTP. However, although NF-κB is essential for the activation of IL-8 transcription, AP-1 appears to contribute to maximal gene expression.

### dTCTP activates NF-κB pathway and inhibition of NF-κB abrogates IL-8 induction by dTCTP

**Fig. 3:**
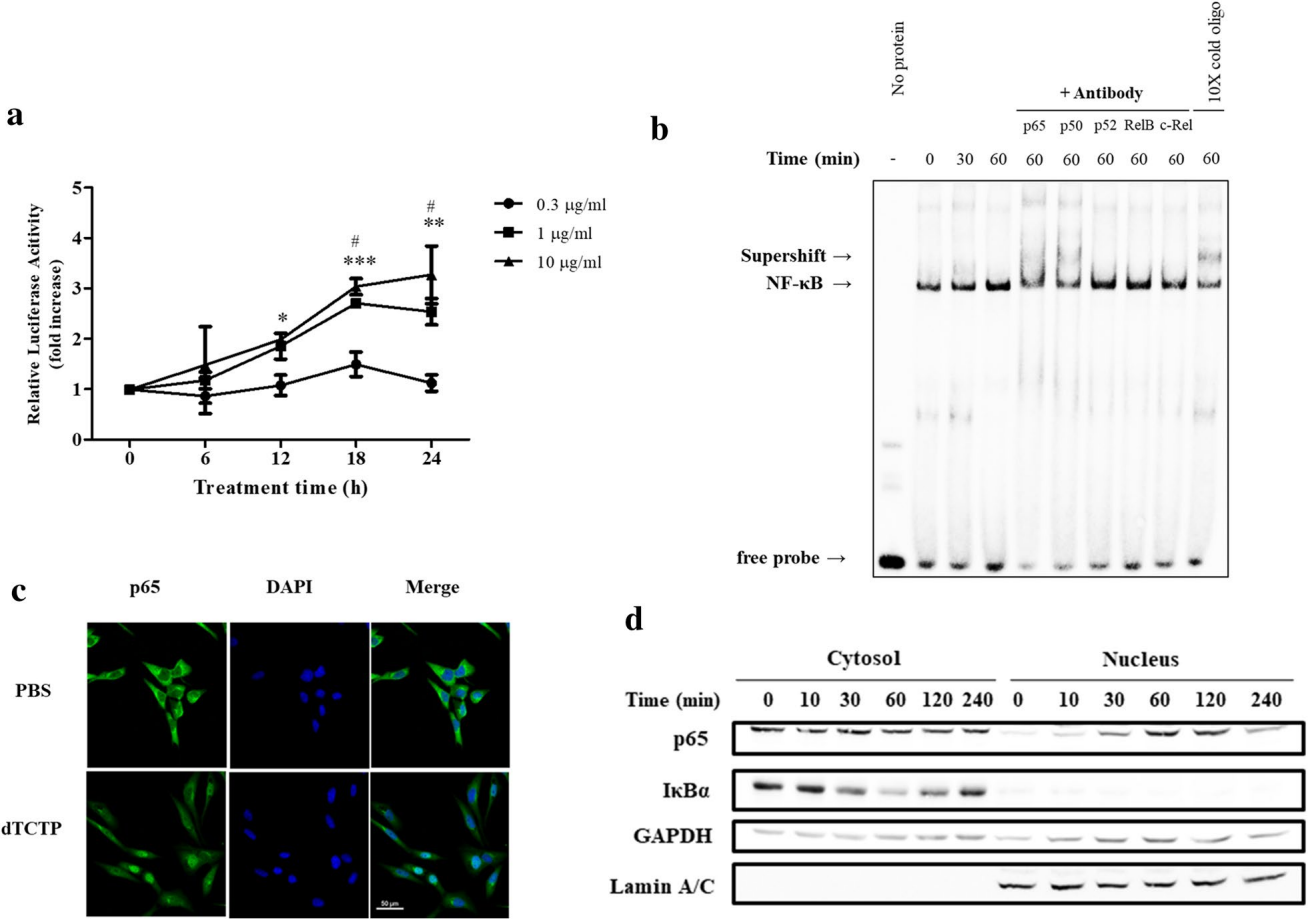
Activation of NF-κB pathway by dTCTP in BEAS-2B cells. **a** NF-κB-driven luciferase activity was measured in dTCTP-treated BEAS-2B cells that were transiently transfected with pNF-κB-Luc. To compensate for transfection efficiency, cells were cotransfected with pRL-TK vector, and the results are presented as a ratio of firefly luficerase to Renilla luciferase. Values represent mean ± SEM, n = 3. ^†^p < 0.05; control vs 1 μg/ml, *p < 0.05, **p < 0.01, ***p < 0.001; control vs 10 μg/ml. **b** Nuclear extracts of BEAS-2B cells were subjected to supershift assays with either no antibody or the indicated antibodies. They were incubated with nuclear extracts for 20 min prior to binding reactions. Arrows, DNA-binding specific complexes. Results are representative of three experiments. **c** BEAS-2B cells were treated with 10 μg/ml of dTCTP for the times indicated. Cells were harvested and nuclear and cytosol fractions were isolated. Each compartment was analyzed by immunoblotting. The experiments were repeated three times. **d** NF-κB nuclear translocation was assessed by immunofluorescence staining for NF-κB p65 (green) in BEAS-2B cells exposed to 10 μg/ml of dTCTP for indicated times. Nucleus was detected by DAPI (blue). Similar results are obtained from three independent experiments and the scale bars were 50 μm

NF-κB represents the nuclear factor-κB and is a transcription factor that regulates gene expression. It is involved in immune and inflammatory mechanisms and regulates cell proliferation, differentiation, death, and tumor formation. It is also known to play an important role in internal signaling in the development of vertebrate animals. As NF-κB seems to play an important player in IL-8 induction by dTCTP, we investigated whether dTCTP activates NF-κB pathway. We first tested the ability of dTCTP to induce luciferase expression using the luciferase reporter plasmid under the control of the NF-κB response element. Figure 3a demonstrates that dTCTP induces NF-κB transcriptional activity. Specifically, the luciferase activity of cells treated with 10 μg/ml of dTCTP for 24 h was increased to 3.3 times that of the control.

The NF-κB protein family includes NF-κB1, NF-κB2, Relish, RelA (p65) RelB, c-Rel, and the Drosophila proteins Dorsal and Dif, which shares the Rel homology domain (RHD). It acts as homodimer or heterodimer on the kb site to regulate transcriptional activity, and the specificity of the response to external stimuli depends on which pair of dimer is formed. To characterize which subunit comprises NF-κB dimer in dTCTP-induced signaling, a supershift assay was performed using antibodies specific for the Rel and NF-κB family members. In Fig. 3b, incubation with p65 or p50 antibodies caused a retarded mobility in DNA–protein complex, suggesting that nuclear extracts from dTCTP stimulated cells contain both p65 and p50 subunits. The result is consistent with the western blotting analysis in Fig. 3c to confirm the translocation of p65 subunit from cytosol to the nucleus. In the experiment, BEAS-2B cells were treated with dTCTP for indicated times and the nuclear compartment was fractionated and blotted for p65 and IκBα expression in each compartment. In the nucleus fraction, p65 was clearly introduced within 30 min of stimulation and degraded after 240 min of stimulation. IκBα begins to be proteolyzed at 30 min and restored to its basal level after 120 min. Mechanistically, IκBα degradation precedes p65 translocation, the time laps between p65 and IκBα are comprehensible. GAPDH and Lamin A/C expression were measured to ensure equal loading and homogeneity of each fraction. In Fig. 3d, localization of NF-κB p65 in cells was visualized using immunofluorescence staining. These data demonstrated that canonical NF-κB pathway was activated in response to dTCTP stimulation.

**Fig. 4:**
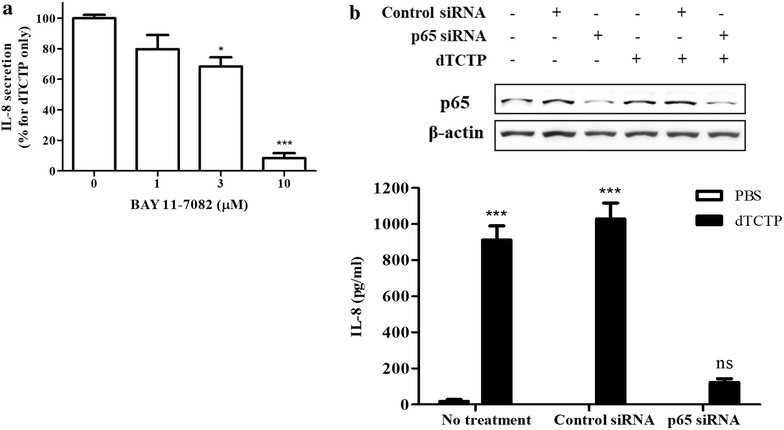
Inhibition of NF-κB blocks IL-8 induction by dTCTP. **a** BEAS-2B cells were pre-incubated with the IκB kinase inhibitor, BAY 11-7082, at the indicated doses for 30 min and then treated with 10 μg/ml of dTCTP for an additional 16–20 h. The resulting IL-8 in the supernatant were analyzed by ELISA. Values represent mean ± SEM (n = 3). *p < 0.05, ***p < 0.001, significantly different from control group. **b** BEAS-2B cells were transfected with control siRNA or p65 siRNA (20 nM) for 24 h as described in the Methods section. After 16–20 h of treatment with or without 10 μg/ml of dTCTP, cell lysates (5 μg) were immunoblotted and probed with p65 or β-actin antibody (upper panel). The supernatant was harvested and the IL-8 content was measured using ELISA (lower panel). Values represent mean ± SEM (n = 3). *ns* not significant from untreated group. ***p < 0.001, significantly different from untreated group

To ensure the importance of NF-κB in IL-8 production triggered by dTCTP, we blocked NF-κB activity before dTCTP stimulation and estimated following IL-8 expression in protein levels (Fig. 4a). BAY11-7082, an irreversible inhibitor of IKKα which is responsible for IκBα degradation, was used to block NF-κB activation process. BEAS-2B cells preincubated with increasing concentrations of BAY11-7082 for 30 min were stimulated with 10 μg/ml of dTCTP for another 16 h. IL-8 proteins in the culture media were measured by sandwich ELISA, which showed reductions in a concentration-dependent manner. The role of NF-κB in IL-8 production was also investigated using p65 siRNA. p65 was efficiently down regulated only in the group transfected with siRNA targeting p65 mRNA (Fig. 4b, upper panel). In the subsequent ELISA revealed that the p65 knockdown group did not show a significant increase in IL-8 after dTCTP treatment (Fig. 4b, lower panel). On the other hand, IL-8 secretion was significantly increased by dTCTP in both transfection-free and control siRNA transfection groups. These results suggest that NF-κB activation is an essential process for the production of IL-8 by dTCTP in BEAS-2B cells.

### MAPKs are involved in dTCTP-induced IL-8 expression

AP-1 is one of the early-identified mammalian transcription factors that activate cell growth, death and differentiation by activating target genes for a variety of external stimuli such as inflammatory cytokines, growth factors and stress. The signaling pathway to control the activity of AP-1 is typically the MAPK pathway. MAPK includes proteins of extracellular signal-regulated kinases (ERKs), c-Jun N-terminal kinases (JNKs) and p38 mitogen-activated protein kinases (p38 MAPK), which share many properties such as activation by phosphorylation of serine and threonine residues, three-step activation pathway and similar substrate recognition sites.

**Fig. 5:**
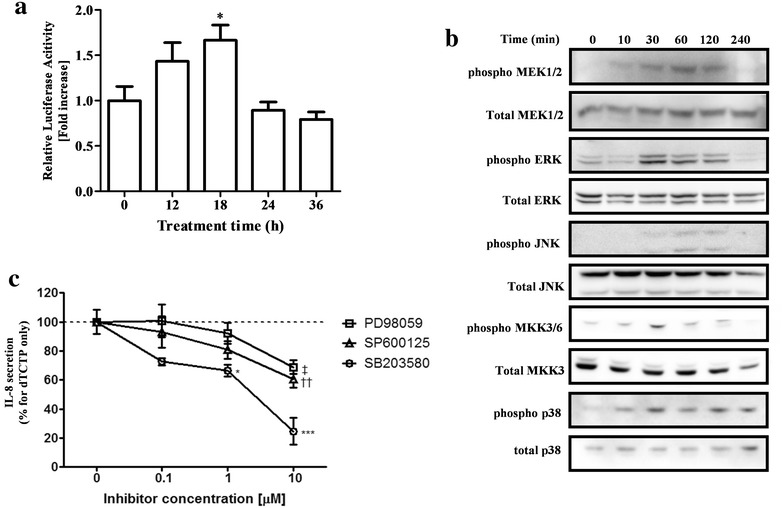
Activation of MAPK pathway by dTCTP in BEAS-2B cells. **a** AP-1-driven luciferase activity was measured in dTCTP-treated BEAS-2B cells that were transiently transfected with pAP-1-Luc. To compensate for transfection efficiency, cells were co-transfected with pRL-TK vector, and the results are presented as a ratio of firefly luciferase to Renilla luciferase. Values represent mean ± SEM (n = 3). *p < 0.05, significantly different from untreated group. **b** BEAS-2B cells were treated with 10 μg/ml of dTCTP for the times indicated. The whole cell lysates were analyzed by immunoblotting. Results are representative of at least three experiments. **c** When BEAS-2B cells were treated with PD98059 (a MEK1 inhibitor), SP600125 (a JNK inhibitor) or SB203580 (a p38 MAPK inhibitor) for 30 min prior to exposure to dTCTP (10 μg/ml), IL-8 production was blocked in a dose-dependent manner. Values represent mean ± SEM, n = 3. ^‡^p < 0.01; control vs PD98059, ^††^p < 0.01; control vs SP600125 and *p < 0.05, ***p < 0.001; control vs SB203580

We first measured the AP-1 transcriptional activity against dTCTP using the inducible AP-1 luciferase vector. Twenty-four hours after transfection of the reporter vector into BEAS-2B cells, dTCTP was treated for the indicated time and luciferase activity was measured. As a result, transcription activity by dTCTP increased up to 1.7-fold (Fig. 5a). We next examined the phosphorylation of MAPKK and MAPK by dTCTP treatment in order to identify the upper MAPK pathway that activates AP-1. In Fig. 5b, MEK1/2 phosphorylation was observed 10 min after treatment with dTCTP, and ERK signal also increased from 30 min, and then decreased after 120 min. In the case of JNK, slight phosphorylation was observed at 60–120 min. MKK3/6 and p38 MAPK were significantly phosphorylated from 30 min and gradually decreased. We used specific inhibitors of MAPK to investigate the extent to which each MAPK contributes to IL-8 production by dTCTP (Fig. 5c). Prior to this experiment, we confirmed that the use of 10 μM of each inhibitor did not affect cell viability or the activity of two other MAPKs (data not shown). Cells treated with inhibitors for 30 min were stimulated with dTCTP and the amount of IL-8 produced after 16 h was measured. All three MAPK inhibitors showed a dose-dependent decrease in IL-8 secretion, among which the p38 inhibitor SB203580 showed the most dramatic reduction. At the highest concentration of 10 μM, IL-8 production was reduced to 69.1% for PD98059, 60.6% for SP600125, and 23.9% for SB203580 compared to the control. From these results, it can be seen that the three MAPKs, particularly the p38 pathway, are involved in the signaling pathway of dTCTP.

These experiments confirmed that p38 MAPK and NF-κB are the major contributors to IL-8 expression by dTCTP. p38 MAPK signaling has been reported to be NF-κB-dependent or independent, depending on the type or duration of stimulation and the cell type examined.

### p38 MAPK is independent of NF-κB activation and increases the stability of IL-8 mRNA

**Fig. 6:**
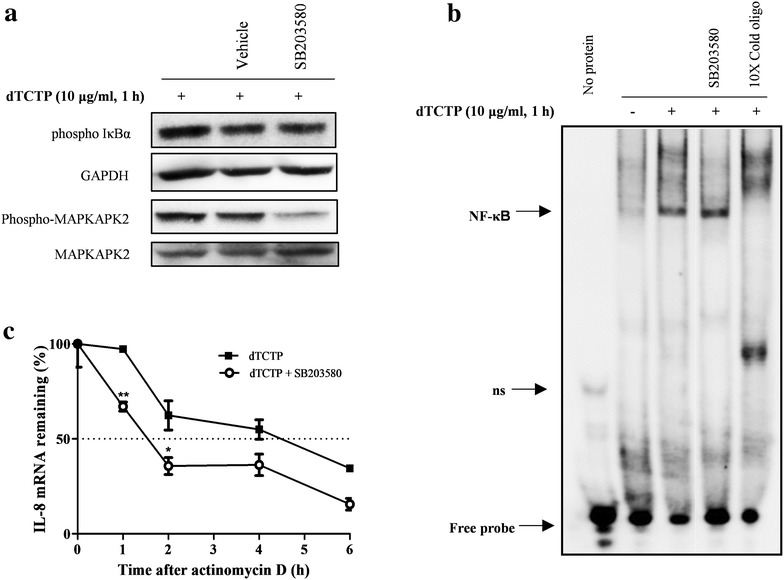
Role of p38 MAPK on IL-8 mRNA stability in BEAS-2B cells. **a** BEAS-2B cells were pretreated with vehicle or SB203580 (10 μM) for 30 min, and stimulated with dTCTP (10 μg/ml) for 1 h. Cell lysates were immunoblotted with the indicated antibodies. **b** Nuclear extracts were obtained from dTCTP (10 μg/ml, 1 h) stimulated cells pretreated with SB203580 (10 μM) for 30 min. Nuclear extracts without stimulation were also prepared as a negative control. They were subjected to EMSA analysis using biotinylated probes and the specificity of the binding reaction was confirmed using 10-molar excess of unlabeled oligos. The results represent three experiments. **c** Real-time PCR analysis of IL-8 was performed to determine remaining mRNA levels. Total RNA was prepared from BEAS-2B cells pre-stimulated with dTCTP (10 μg/ml) for 6 h followed by treatment with or without SB203580 (10 μM) for 30 min, and incubated with actinomycin D (1 μg/ml) for 0, 1, 2, 4 or 6 h. IL-8 mRNA levels of dTCTP-treated group without actinomycin D (0 h) were set as 100% IL-8 mRNA and relative percentages were calculated with respect to the 100% dTCTP control at other time points. Values are mean ± SEM (n = 3). *p < 0.05, **p < 0.01; significantly different from dTCTP treated group

We then investigated whether dTCTP-induced NF-κB activation is affected by the p38 MAPK inhibitor. After incubation for 30 min with SB203580, cells were stimulated with dTCTP for 1 h and the changes in IκBα phosphorylation were detected by immunoblotting (Fig. 6a). The results showed that inhibition of p38 MAPK did not affect IκBα phosphorylation. Next, we performed EMSA to determine whether p38 MAPK affects the DNA binding activity of NF-κB. Figure 6b shows, increased DNA–protein complexes in samples treated with dTCTP did not change upon treatment with SB203580.

One of the unique functions of p38 MAPK is its role in regulating mRNA stability of proinflammatory cytokines. Most inflammatory cytokine mRNA derived from normal cells has a short half-life due to AU-rich elements (AREs) of 3′ untranslated region. It is well known that IL-8 mRNA, which contains ARE, induces chronic inflammation by inducing phosphorylation of ARE binding protein tristetraprolin (TPP) in response to various stimuli due to activation of p38 MAPK, thereby preventing mRNA from decaying. We therefore investigated whether dTCTP-activated p38 MAPK potentiates IL-8 expression by modulating mRNA stability (Fig. 6c). Cells treated or not treated with inhibitor were stimulated with dTCTP for a period of time and incubated with actinomycin D and the residual mRNA was measured over time. In the dTCTP-treated group, the half-life of IL-8 mRNA was estimated as 4.5 h. However, when the activity of p38 MAPK was inhibited, half-life of IL-8 mRNA decreased by 65.5% to 1.5 h. Thus, in our system using dTCTP, we conclude that p38 MAPK potentiates IL-8 gene expression by stabilizing IL-8 mRNA without affecting the NF-κB pathway.

## Discussion

In early studies using basophils isolated from allergic donors, HRF was thought to work in an IgE-dependent manner because HRF provoked histamine release only in some basophils [15, 16]. These differences led to the classification of IgE by IgE+/IgE−. However, in subsequent studies, it was reported that HRF enhanced anti-IgE antibody-induced histamine release in basophils regardless of type of cell surface IgE, and that the IgE antibody and HRF did not bind directly [17, 18].

Allergic response through antigen-IgE-FcεRI axis is well-known. However, it has been shown that without multivalent hapten, certain populations of monomeric IgE can also activate FcεRI aggregation and deliver the subsequent signal transduction events [19]. IgE molecules have a broad spectrum of mast cell activation potential [20], and the IgEs categorized as highly cytokinergic tend to bind better to autoantigen such as HRF. This means that the results can vary widely depending on the IgE molecules used in the experiment. In this context, we can comprehensively understand the conflicting results of the relationship between HRF and IgE. As the new function of IgE was established, Kawakami et al. reassessed the relationship between IgE and HRF and suggested that IgE and IgG are HRF receptors that have been sought for a long time [21]. Using a large panel of IgE and IgG, they demonstrated that HRF binds to the Fab sites of a subset of IgE and IgG. Peptides that interfere with HRF-Ig binding have been shown to inhibit mast cell activation and suppress passive cutaneous anaphylaxis and mast cell dependent airway inflammation. Furthermore, they revealed that pulmonary inflammation induced by nasal administration of HRF in naïve mice occurs via B cells, mast cells and FcεRIα and Fcγ using genetically deficient mice.

Although the primary involvement of IgE and Fc epsilon receptor in the inflammatory response by HRF has been clearly demonstrated by the ‘knock out’ system, we cannot dispense with the possibility of another HRF-specific receptor other than IgE for the following reasons. First of all, in HRF-responsive basophils in which HRF-mediated direct histamine release was found, phosphorylation of intercellular signaling molecules did not accompany FcεRIγ phosphorylation [22]. Next, HRF and anti-IgE showed different results in response to the treatment of a pharmacologic inhibitor, rottlerin, implying that they induce histamine release through different signal transduction pathway [23]. Anti-IgE-induced histamine release was not affected by rottlerin, but HRF-mediated histamine release increased, demonstrating that HRF signaling is similar to but not entirely equivalent to that of IgE. Although we cannot rule out the possibility that this is due to qualitative and quantitative differences in the signals induced by Highly cytokinergic (HC) IgE and IgE + Ag, signals transmitted to cells are very similar except for some points because they act through essentially the same receptor [24]. Finally, HRF caused IL-8 and GM-CSF cytokine secretion in AML14-3D10 [25] and BEAS-2B [6] cell lines that do not express detectable FcεRIα (see Additional file 2: Figure S2). Functional receptors for IgE have been reported to be expressed in bronchial epithelial cells in some asthmatic patients, which implies an important message that the expression repertoire of membrane protein changes as asthmatic lesions progress [26, 27]. However, we confirmed the absence of high-affinity IgE receptor in the BEAS-2B cell line even after stimulating the cells with dTCTP. Furthermore, the report that only dimeric TCTP binds to the cell surface of BEAS-2B suggested the presence of other receptor besides FcεRI [5]. In this respect, we sought to obtain clues for the receptor by identifying signaling molecules involved in IL-8 expression by HRF in BEAS-2B cells, where the receptor is presumed to be present.

The regulation of the IL-8 gene expression has been well characterized [13, 14, 28, 29]. The promoter region of IL-8 gene is restrained by several mechanisms, so that the protein is barely detectable in normal physiology. The restrained promoter should be derepressed through following mechanisms: (1) altered function of NF-κB-repressing factor (NRF) as a coactivator which is originally involved in basal silencing of IL-8 gene in resting cells, (2) replacement of octamer-1 (Oct-1) with CAAT/enhancer-binding protein (C/EBP) which has binding site on the complementary strand of the octamer motif (5′-ATTTGCAT-3′), and (3) recruitment of CREB-binding protein (CBP)/p300, followed by hyperacetylation of histones and chromatin remodeling. When the promoter is activated by various stimuli such as TNFα and IL-1β, IL-8 protein expression increases from tens to hundreds of fold. In previous studies on the mechanism of IL-8 expression, NF-κB and AP-1 have been shown to play pivotal roles [30–36], but the specific mechanism depends on the type of stimulus and cell used.

In our experimental conditions, dTCTP induced phosphorylation of ERK, JNK, and p38 MAPK and activated canonical NF-κB pathway. We confirmed that those pathways were specific for dTCTP in the following two experiments. First, only dTCTP phosphorylated ERK and IκBα and degraded IκBα, while mTCTP had no such activity (see Additional file 3: Figure S3A). In addition, dTBP2 peptide, which was found to bind to dTCTP and to inhibit its cytokine-like activity [37], suppressed dTCTP-induced phosphorylation of ERK and IκBα (see Additional file 3: Figure S3B). Notably, the mutation in the κB site of the IL-8 promoter abrogated dTCTP-induced promoter activity, suggesting that the transcriptional activation by NF-κB was indispensable for IL-8 production (Fig. 2). However, it seemed that NF-κB alone could not develop maximal IL-8 expression by dTCTP. This was inferred from the fact that IL-8 production was induced by 5.1-fold (see Additional file 4: Figure S4), although there was no significant increase in NF-κB luciferase activity in Fig. 3a when the dose of dTCTP was increased from 1 to 10 μg/ml. These findings suggest the additional mechanism other than transcriptional activation by NF-κB is required for the maximal IL-8 expression induced by dTCTP. We demonstrated that the promoter activity decreased significantly when the AP-1 binding site was mutated, implying its role in IL-8 transcriptional activation (Fig. 2c). It was also confirmed that dTCTP activated MAPKs, known as the upstream molecule of AP-1 dimer. One of the greatest hallmarks of IL-8 is that it exists at a very low level in the resting state, but the expression level increases dramatically in response to the stimulation. This dramatic increase is known to be mainly regulated at the mRNA level, but the half-life of IL-8 mRNA is reported to be very short, about 1.5 h [38, 39]. In this respect, the threefold increase in the half-life of mRNA by p38 MAPK may have a significant impact on IL-8 production induced by dTCTP (Fig. 6c). These findings suggest that NF-κB pathway is essential, but not sufficient for IL-8 expression and that the additional transcriptional activation by AP-1 and post-transcriptional regulation such as mRNA stabilization by p38 MAPK are required for the maximal IL-8 expression by dTCTP.

Several major research groups have reported on signaling molecules activated by HRF. Langdon and her colleagues reported a negative correlation between SHIP-1 level and histamine releasability in response to HRF [40]. Also, they showed phosphorylation events of Syk, Akt (Thr308), MEK (Ser217/221) and ERK (Thr202/Tyr204) by HRF [22]. In another study that identified the role of HRF as a B cell stimulator, Kang et al. [41] showed that HRF increased NF-κB activity in splenic B cells, particularly when co-incubated with anti-CD40 antibody. And recently, Xiao et al. who studied the role of extracellular TCTP in colorectal cancer progression, reported that extracellular TCTP promotes CRC progression and liver metastasis through the Cdc42/JNK/MMP9 axis [42]. In this study, it was stated that signaling molecules such as phospho Akt1 (Ser473), phospho MEK1 (SER217/221), phospho ERK1/2 (Thr202/Tyr204), phospho p38 MAPK (Thr180/Tyr182), phospho Stat3 (Tyr705), and phospho NF-κB p65 (Ser536) were not altered by extracellular TCTP: This study is very interesting as the first report that extracellular TCTP causes the invasion and metastasis of tumors previously known as the function of intracellular TCTP, but there is no mention of whether the material used is a dimer or monomer.

These reports provide valuable information. However, in the interpretation of the experimental results, we must consider not only the cell type used in each experiment but also the method by which the recombinant protein was produced and its concentration used. We performed this study with the highest concentration of 10 μg/ml based on the concentration used in BEAS-2B in the previous report [5, 6]. Most of the previous studies using recombinant HRF protein were carried out at concentrations below 10 μg/ml. Notably, HRF directly enhanced B cell proliferation at concentrations of less than 1 μg/ml, and could activate B cell growth even at 100-fold lower concentrations, especially in the presence of anti-CD40 Ab [41]. If we can rule out other experimental factors, B cells can be considered the most sensitive of the HRF target cells. Recombinant proteins were expressed in Sf9 insect cells or *E. coli* system, and a vector with the entire HRF sequence was used in most cases. Analysis of the purified recombinant protein on SDS-PAGE showed some degree of spontaneous dimerization of HRF. However, we have established a system for producing dTCTP by inducing intermolecular disulfide bonds between exposed cysteine residue by truncating the N-terminal sequence to produce higher proportions of dimers. Mutants that disrupted disulfide bonds by replacing cysteine with alanine did not produce dimers and failed to induce inflammation when administered intranasally [5, 21]. However, it is not yet clear which form of dimer is found in vivo, and the answer could hold the key to HRF research.

In summary, HRF appears to function in the orchestration of various cells at higher stages of late phase response. It is also found in normal serum, but for some reason it does not cause inflammation. But once switched on, bioactive HRF causes extensive downstream signaling as a multi-modulator of the immune system. Depending on the mode of action in each target cell, the response or subsequent signaling caused by HRF will be different. Our study demonstrated that in BEAS-2B cells, dTCTP induces IL-8, an inflammatory cytokine, through MAPK and NF-κB signaling. NF-κB activation was essential for IL-8 production by HRF, and for maximal production, AP-1 activation by MAPKs and IL-8 mRNA stabilization by p38 MAPK were also required. Identifying kinases and adapter molecules upstream of this signaling event will be an important clue for receptor classification and is currently under investigation.

## Additional files

**Additional file 1: Figure S1.** The binding reaction in EMSA assays is specific for NF-κB and AP-1. Nuclear extracts were prepared from the BEAS-2B cells stimulated with or without 10 μg/ml of dTCTP for 60 min. EMSA was performed according to the Methods using biotinylated probes for (A) NF-κB and (B) AP-1. To examine the specificity of the binding reactions, tenfold molar excess of unlabeled wild-type oligo or tenfold molar excess of unlabeled mutant oligo was added to the binding mixture.

**Additional file 2: Figure S2.** Lack of FcεRIα expression in BEAS-2B cells. FcεRIα expression was determined by immunoblotting in BMMC and BEAS-2B cells. For BEAS-2B, 10 μg/ml of dTCTP was treated for the indicated times and the change in protein expression were measured. BMMC was used as a positive control for cells expressing FcεRIα.

**Additional file 3: Figure S3.** ERK and NF-κB pathways are specific for dTCTP. (A) Only dTCTP, not mTCTP, activated ERK and NF-κB pathways in BEAS-2B cells. Cells were stimulated with full-length TCTP (20 μg/ml) or Del-N11TCTP (10 μg/ml) or for 1 h. The whole cell lysates were analyzed by immunoblotting. (B) dTBP2 suppress dTCTP-induced ERK and NF-κB signaling pathways in BEAS-2B cells. PBS or dTBP2 was pre-incubated with dTCTP for 10 min and treated to BEAS-2B cells. After 1 h, cells were harvested and the whole cell lysates were analyzed by immunoblotting.

**Additional file 4: Figure S4.** Dose-denpendent IL-8 release by dTCTP. BEAS-2B cells were stimulated with the indicated doses of dTCTP (0–10 μg/ml) and incubated for 16 h. The IL-8 protein released into the supernatant was measured using a sandwhich ELISA kit. The relative percentage was calculated by setting the maximum value of IL-8 to 100%.
