# Supplementary figures and images for: Dimerized translationally controlled tumor protein increases interleukin-8 expression through MAPK and NF-κB pathways in a human bronchial epithelial cell line
Source: Cell Biosci. 2018 Feb 20;8:13. doi: 10.1186/s13578-018-0214-6 (PMC5819651; doi:10.1186/s13578-018-0214-6)

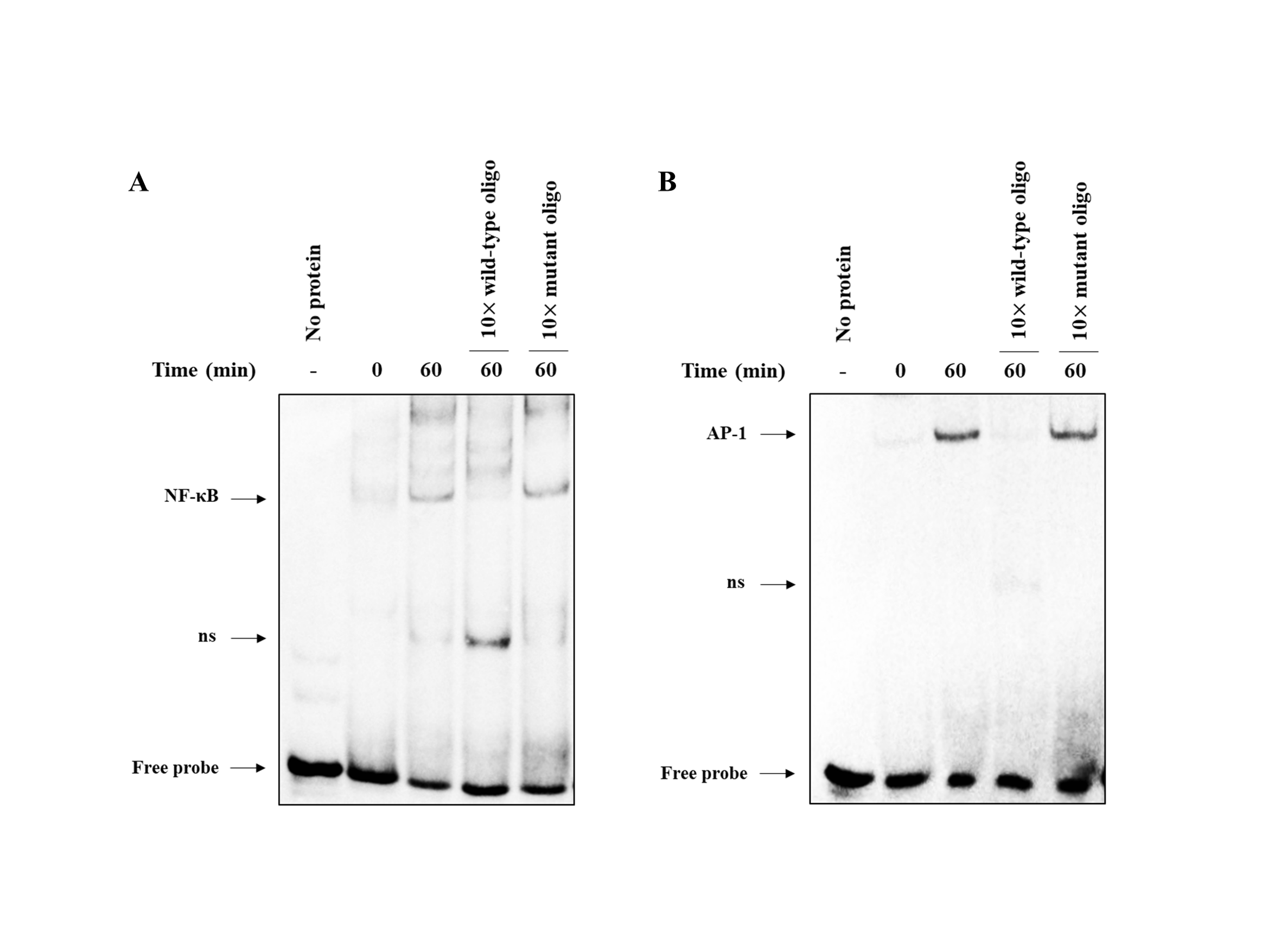

Supplement: Supplementary file 1 — Additional file 1: Figure S1. The binding reaction in EMSA assays is specific for NF-κB and AP-1. Nuclear extracts were prepared from the BEAS-2B cells stimulated with or without 10 μg/ml of dTCTP for 60 min. EMSA was performed according to the Methods using biotinylated probes for (A) NF-κB and (B) AP-1. To examine the specificity of the binding reactions, tenfold molar excess of unlabeled wild-type oligo or tenfold molar excess of unlabeled mutant oligo was added to the binding mixture. [file 13578_2018_214_MOESM1_ESM.tif]

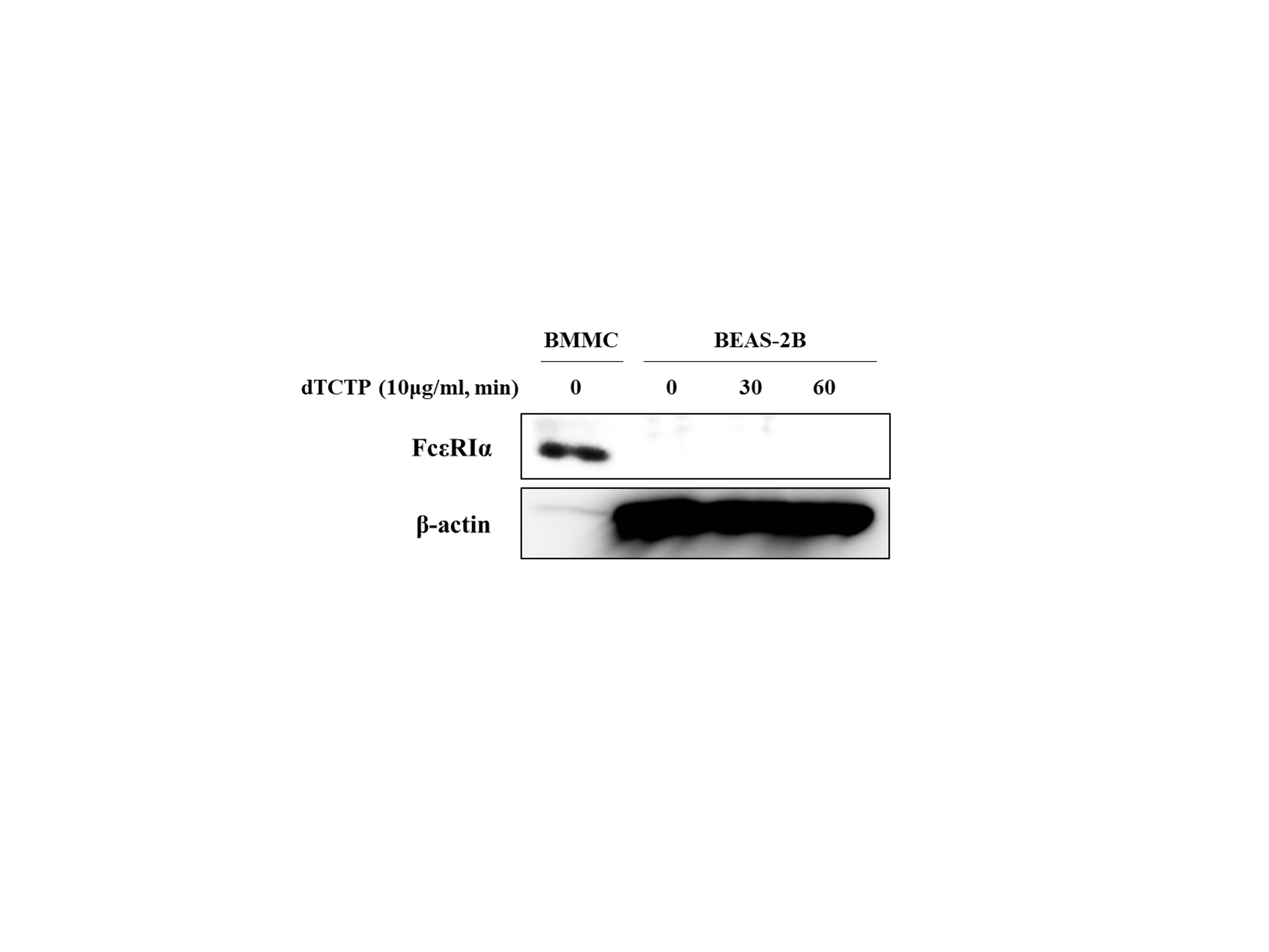

Supplement: Supplementary file 2 — Additional file 2: Figure S2. Lack of FcεRIα expression in BEAS-2B cells. FcεRIα expression was determined by immunoblotting in BMMC and BEAS-2B cells. For BEAS-2B, 10 μg/ml of dTCTP was treated for the indicated times and the change in protein expression were measured. BMMC was used as a positive control for cells expressing FcεRIα. [file 13578_2018_214_MOESM2_ESM.tif]

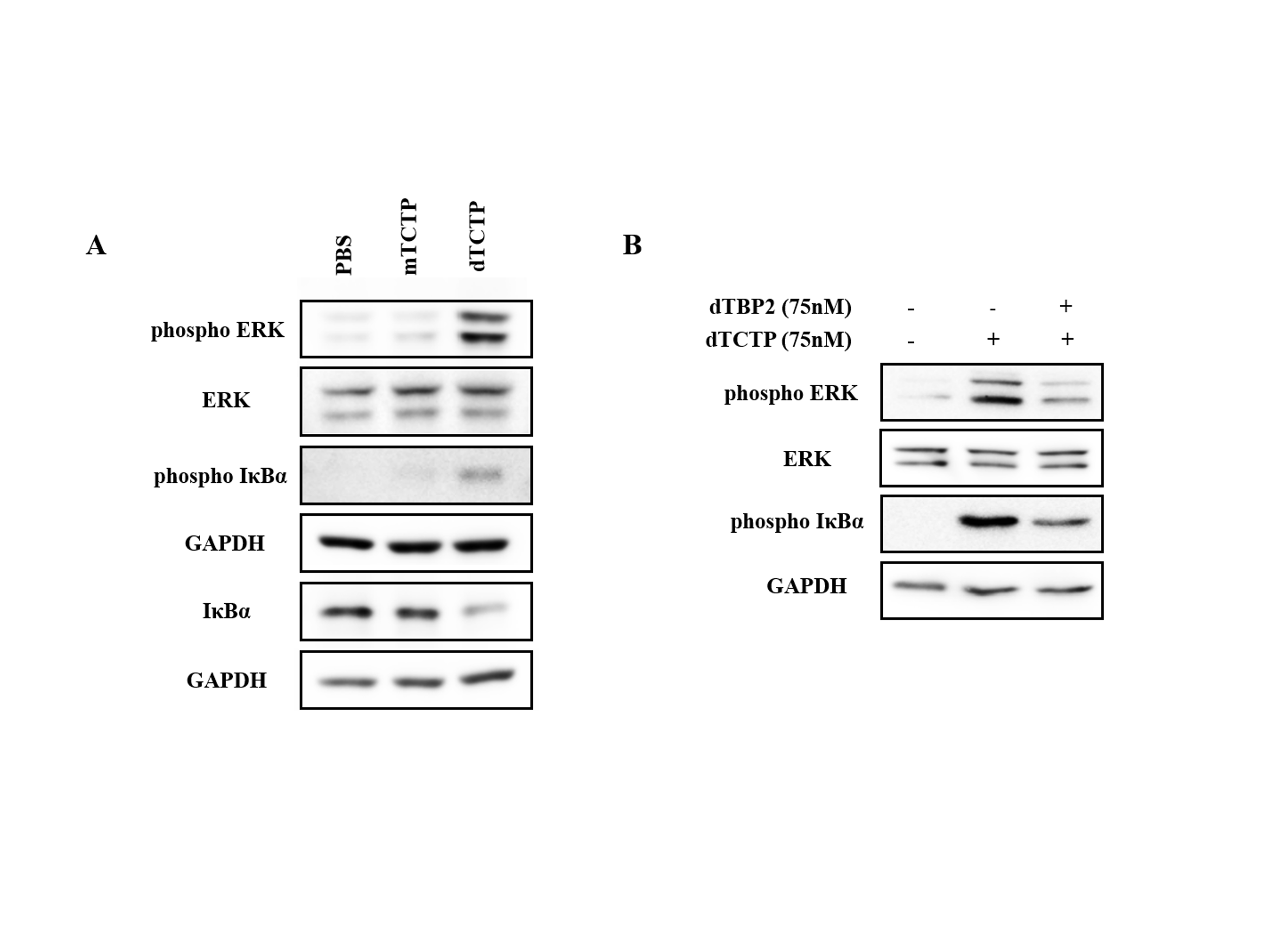

Supplement: Supplementary file 3 — Additional file 3: Figure S3. ERK and NF-κB pathways are specific for dTCTP. (A) Only dTCTP, not mTCTP, activated ERK and NF-κB pathways in BEAS-2B cells. Cells were stimulated with full-length TCTP (20 μg/ml) or Del-N11TCTP (10 μg/ml) or for 1 h. The whole cell lysates were analyzed by immunoblotting. (B) dTBP2 suppress dTCTP-induced ERK and NF-κB signaling pathways in BEAS-2B cells. PBS or dTBP2 was pre-incubated with dTCTP for 10 min and treated to BEAS-2B cells. After 1 h, cells were harvested and the whole cell lysates were analyzed by immunoblotting. [file 13578_2018_214_MOESM3_ESM.tif]

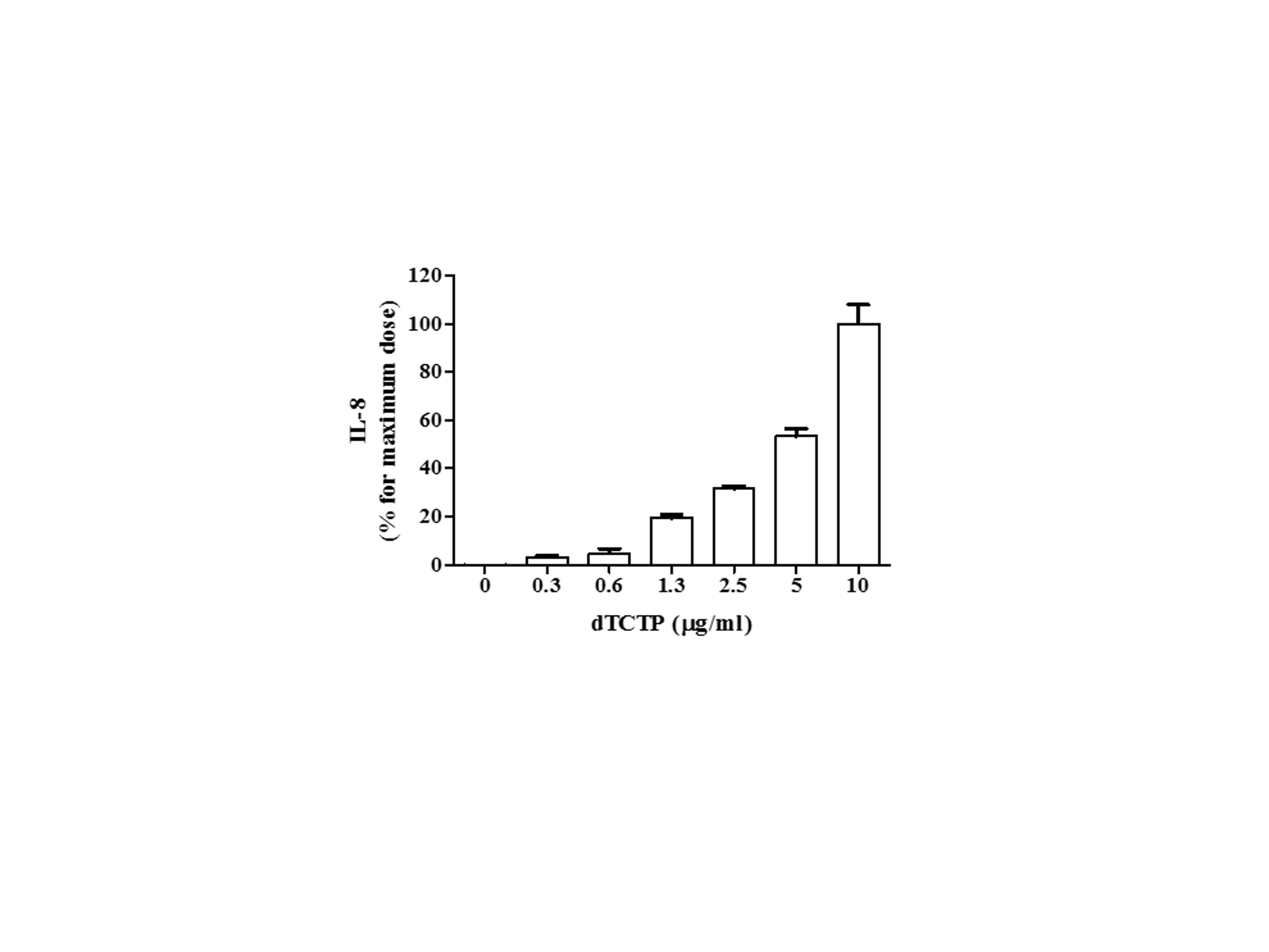

Supplement: Supplementary file 4 — Additional file 4: Figure S4. Dose-denpendent IL-8 release by dTCTP. BEAS-2B cells were stimulated with the indicated doses of dTCTP (0–10 μg/ml) and incubated for 16 h. The IL-8 protein released into the supernatant was measured using a sandwhich ELISA kit. The relative percentage was calculated by setting the maximum value of IL-8 to 100%. [file 13578_2018_214_MOESM4_ESM.tif]
